# Renal Vein Thrombosis in a Patient with Sickle Cell Disease: A Rare Diagnostic Challenge

**DOI:** 10.51894/001c.166001

**Published:** 2026-07-31

**Authors:** Zohaib Khan, Abdallah Almawazreh, Abu Fazal Shaik, Kamal Khalil

**Affiliations:** 1 DMC - Sinai Grace

## 116

### Background

Renal vein thrombosis (RVT) is an uncommon diagnosis in adults and is rarely reported in patients with sickle cell disease (SCD). Diagnostic complexity arises from significant overlap between RVT and SCD-related vaso-occlusive crises, as both may present with abdominal or flank pain, hematuria, and renal dysfunction. SCD is a chronic hypercoagulable state associated with increased risk of venous thromboembolism, including thrombosis at atypical sites. Pathophysiologic mechanisms such as sickled erythrocyte–mediated vascular occlusion, endothelial dysfunction, inflammation, and infection contribute to thrombus formation. Laboratory abnormalities including elevated D-dimer, lactate dehydrogenase, and leukocytosis are common in SCD even in the absence of thrombosis, limiting specificity. Imaging is therefore essential, with contrast-enhanced computed tomography (CT) or magnetic resonance imaging providing confirmation.

### Case Presentation

A 39-year-old woman with HbSS SCD, poorly controlled type II diabetes mellitus (HbA1c 9.9%), and recurrent urinary tract infections presented with severe lower abdominal and right flank pain, dysuria, dark urine, nausea, and vomiting. She had recently been discharged after treatment for vaso-occlusive crisis and pyelonephritis but was unable to obtain medications. On presentation, blood pressure was 157/95 mmHg, heart rate 96 bpm, and she was afebrile. Examination revealed suprapubic and right costovertebral angle tenderness. Laboratory evaluation showed hemoglobin 10.6 g/dL, creatinine 0.87 mg/dL, and pyuria. CT abdomen and pelvis demonstrated mild left-sided pyelonephritis and an interval non-obstructing left RVT. She was treated with intravenous ceftriaxone and a heparin infusion, later transitioned to enoxaparin and subsequently apixaban 5 mg twice daily. Hydroxyurea was temporarily held. Pain was managed with opioids. Symptoms improved, and she was discharged on oral trimethoprim-sulfamethoxazole and anticoagulation.

### Discussion

This case highlights the diagnostic complexity of RVT in SCD, where overlapping symptoms can obscure diagnosis. Infection, inflammation, and hypercoagulability likely contributed. Contrast-enhanced CT remains the most reliable tool for detection2. Early anticoagulation prevents thrombus extension and preserves renal function. Clinicians should maintain a high index of suspicion for RVT in SCD patients with persistent or atypical flank pain despite standard crisis therapy.

### Conclusion

RVT should be considered in SCD patients with refractory flank pain and urinary symptoms. Timely imaging and multidisciplinary management are essential to optimize outcomes.

